# Synthesis of the fluorescent amino acid *rac*-(7-hydroxycoumarin-4-yl)ethylglycine

**DOI:** 10.3762/bjoc.6.69

**Published:** 2010-06-24

**Authors:** Manfred Braun, Torsten Dittrich

**Affiliations:** 1 Institute of Organic and Macromolecular Chemistry, University of Düsseldorf, Universitätsstr. 1, D-40225 Düsseldorf, Germany

**Keywords:** alkylation, coumarin, fluorescent probe, glycine, protecting group

## Abstract

The hydrochloride of the racemic amino acid (7-hydroxycoumarin-4-yl)ethylglycine, a versatile fluorescent probe in proteins, has been synthesized in five steps from commercially available (7-hydroxycoumarin-4-yl)acetic acid. The key step involves the alkylation of a glycine–enolate equivalent.

## Introduction

**Figure 1:**
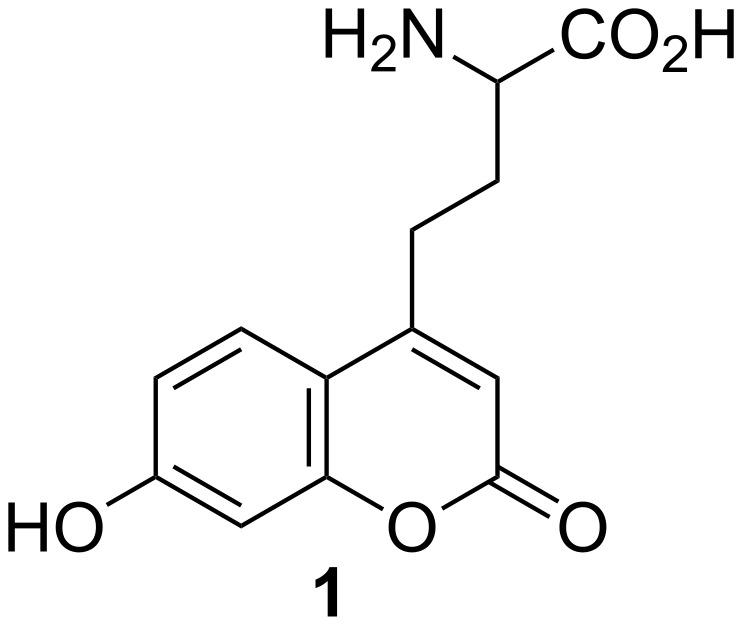
Fluorescent amino acid (7-hydroxycoumarin-4-yl)ethylglycine (**1**).

The incorporation of non-natural fluorescent amino acids into proteins has developed into a potent tool for the investigation of proteins with regard to their structure, function and interaction with substrates [1–3]. Once in vitro and in vivo biochemical methods for the inclusion of non-natural amino acids had been successfully developed [4–9], an easy synthetic access to amino acids with suitable fluorophoric groups that provide tailor-made spectroscopic properties became the next crucial step. The requirements of a suitable fluorescent probe are largely met by (7-hydroxycoumarin-4-yl)ethylglycine (**1**) (Figure 1), which not only displays a relatively large Stokes shift but also a fluorescence that is sensitive to both pH and solvent polarity [10–11]. Although a straightforward, short approach to the L-amino acid **1** has been reported [10], its isolation and purification was found to be difficult and tedious: According to the described protocol, the isolation of final product **1** required preparative reversed-phase HPLC. Unfortunately, this procedure, despite the elegant concept of the synthetic route, is not applicable for the provision of larger quantities of product. As a result we were interested in developing an alternative route to racemic amino acid **1**.

## Results and Discussion

**Scheme 1:**
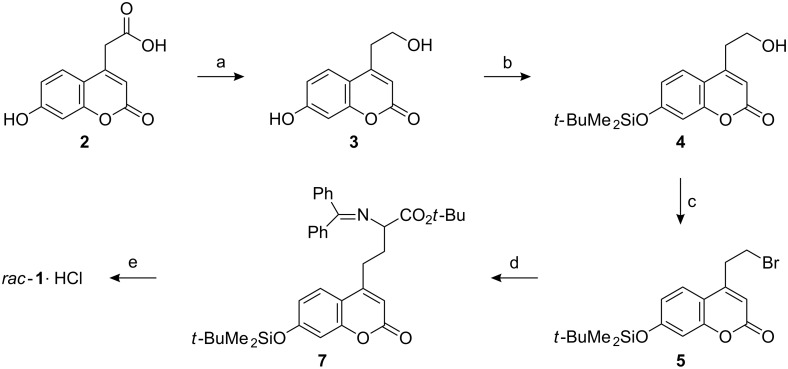
Synthetic route to racemic amino acid **1**·HCl. Reagents and conditions: a) BH_3_·SMe_2_, THF, 0 °C to r. t., 59%; b) *t*-BuMe_2_SiCl, NaH, THF, r. t., 59%; c) CBr_4_, PPh_3_, CH_2_Cl_2_, 0 °C to r. t., 79%; d) Ph_2_C=N-CH_2_-CO_2_*t*-Bu **6**, *n*-BuLi, THF, −78 °C to r. t., 67%; e) HCl, H_2_O, r. t., 60%.

The synthesis outlined in Scheme 1 started from coumarinyl acetic acid **2** which is commercially available or, alternatively, readily accessible [12] through a Pechmann condensation [13] from resorcinol and diethyl acetonedicarboxylate. First, the acid **2** was reduced to the alcohol **3** with borane. The protocol described in the literature [12] was modified, and the reducing agent borane–tetrahydrofuran replaced by the less expensive borane–dimethyl sulfide. This also led to a slight increase in yield (59%). For the planned selective protection of the phenolic hydroxyl group, the *tert*-butyl(dimethyl)silyl group [14] was chosen. Thus, coumarin **3** was treated with one equivalent of sodium hydride to generate the phenolate by selective deprotonation without abstraction of the proton from the primary alcohol moiety. Subsequent treatment with *tert*-butyl(dimethyl)silyl chloride gave alcohol **4** in 59% yield after removal of the doubly silylated side product the formation of which could not be completely avoided. By means of an Appel reaction [15], the alcohol **4** was converted into the bromide **5** in 79% yield. In the following key-step, alkylation of a glycine enolate with the primary bromide **5** was anticipated. Indeed, its use as the electrophilic component in the coupling with the benzophenone-derived imine of *tert*-butyl glycine **6**, which functioned, after deprotonation, as a synthetic equivalent of a glycine enolate synthon, led to the imine **7** in 67% yield. Under the aprotic conditions of the alkylation protocol, the silyl protecting group turned out to be stable, a fact which facilitated the purification of the imine **7**. It is an obvious idea to apply the established protocols for the enantioselective alkylation of ester **6** under phase transfer catalysis [16]. However, only an insignificant enantiomeric excess was observed in the alkylation product **7** when a representative protocol was applied [17]. Finally, the three protecting groups, the *tert*-butyl ester, the imine, and the silyl ether, were removed in a single step by hydrolysis with hydrochloric acid. After repeated extractions with diethyl ether, lyophylization of the acidic aqueous solution gave the hydrochloride of the racemic amino acid **1** as a colorless crystalline material, whose spectroscopic data were identical to those reported in the literature [10].

In summary, a straightforward synthetic route has been devised that leads to the colorless, crystalline hydrochloride of the racemic amino acid **1** in 11% and 19% overall yield starting from commercially available coumarinyl acetic acid **2** or the known alcohol **3**, respectively. The fact that the product obtained is a racemic mixture might be considered a drawback: However, it has been proven for a related non-natural amino acid [8], that in the incorporation into a protein during translation on the ribosome, the L-enantiomer is accepted exclusively from the racemic mixture. Thus, one can assume that, for in vivo incorporation, the amino acid **1** does not have to be enantiomerically pure. Nevertheless, the elaboration of an enantioselective route is ongoing in our laboratory.

## Experimental

General: Melting points (uncorrected) were determined with a Büchi 540 melting point apparatus. NMR spectra were recorded with a Bruker DXR 500 spectrometer. Mass spectra were recorded on an ion-trap API mass spectrometer Finnigan LCQ Deca (ESI), triple-quadrupole-mass spectrometer Finnigan TSQ 7000, and sector field mass spectrometer Finnigan MAT 8200 (EI, 70 eV). Elemental analyses (C, H, N) were performed on Perkin-Elmer 2400 series II at the Institute of Pharmaceutical Chemistry (University of Düsseldorf). High resolution mass spectra were obtained with a Bruker FT-ICR APEX III (7.0 T) (ESI) at the University of Bielefeld. Column chromatography was performed with Fluka silica gel 60 (230–400 mesh) and thin layer chromatography was carried out by using Merck TLC Silicagel 60F_254_ aluminium sheets. Tetrahydrofuran (THF) was freshly distilled from sodium/benzophenone under a nitrogen atmosphere, and dichloromethane was freshly distilled from calcium hydride.

**7-Hydroxy-4-(2-hydroxyethyl)-2*H*-chromen-2-one (3):** A 500 mL flask was equipped with a magnetic stirrer, a pressure-equalizing dropping funnel closed with a septum, and a connection to a combined nitrogen/vacuum line. The flask was charged with carboxylic acid **2** (8.95 g, 40.68 mmol), and the air in the flask was replaced by nitrogen. Dry THF (150 mL) was added and the solution was cooled to 0 °C. Through the dropping funnel, a 10 M solution of borane–dimethyl sulfide (12 mL, 120 mmol) in THF, diluted with 100 mL of dry THF was added dropwise over 60 min at 0 °C. Stirring was continued at r. t. for 24 h. After cooling to 0 °C, water (10 mL) was cautiously added. The mixture was concentrated in a rotary evaporator and the residue treated with ethyl acetate (200 mL) and water (200 mL). The organic layer was separated and the aqueous phase extracted with two 150 mL portions of ethyl acetate. The combined organic layers were dried with sodium sulfate. After the solvent had been removed, the viscous residue was dissolved in acetone and purified by column chromatography with acetone/ethyl acetate (1:4) to give 5.03 g (59%) of a yellowish solid product that was identical with compound **3** according to its ^1^H-NMR spectrum [12]. ^13^C NMR (DMSO-*d*_6_, 125 MHz): 34.53 (*C*H_2_CH_2_OH), 59.41 (CH_2_*C*H_2_OH), 102.28 (C-8), 110.31 (C-4a), 111.46 (C-3), 112.78 (C-6), 126.44 (C-5), 154.58 (C-8a), 155.02 (C-4), 160.26 (C-7), 160.92 (C-2).

**4-(2-Hydroxyethyl)-7-(*t*-butyldimethylsilyloxy)-2*H*-chromen-2-one (4):** A 50 mL two-necked flask equipped with a magnetic stirrer and a connection to a combined nitrogen/vacuum line was charged with alcohol **3** (385 mg, 1.87 mmol) and closed with a septum. The air in the flask was replaced by nitrogen, and dry THF (10 mL) was injected by syringe. The septum was removed for a short time, and a 60% suspension of sodium hydride in mineral oil (74.8 mg, 1.87 mmol) added in one portion. The flask was closed again, and the mixture was stirred for 30 min at r. t. *t*-Butyl(dimethyl)chlorosilane (281 mg, 1.87 mmol) was added and stirring continued at r. t. until TLC showed the absence of alcohol **3** (approximately 1 h). The mixture was poured into ice and water and extracted three times with ethyl acetate (total volume 100 mL). The combined organic layers were washed with 2 N hydrochloric acid (20 mL) and dried with sodium sulfate. After the solvent had been removed in a rotary evaporator, the oily residue was submitted to column chromatography with ethyl acetate to give 353 mg (59%) of an oily, yellowish product **4** that subsequently crystallized. *R*_f_ 0.72; m. p. 100.5 °C. IR (KBr): 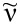 = 3423, 2958, 2928, 2894, 2856, 1679, 1613, 1410, 1292, 1192, 1143, 997, 846, 782 cm^−1^. ^1^H NMR (CDCl_3_, 500 MHz): δ = 0.29 [s, 6H, Si(CH_3_)_2_], 0.98 [s, 9H, C(CH_3_)_3_], 2.99 (t, *J* = 6.3 Hz, 2H, C*H*_2_CH_2_OH), 3.98 (t, *J* = 6.31 Hz, 2H, CH_2_C*H*_2_OH), 6.20 (s, 1H, 3-H), 6.78 (m, 2H, 6-H and 8-H), 7.51 (d, *J* = 9.5 Hz, 1H, 5-H). ^13^C NMR (CDCl_3_, 125 MHz): δ = −4.0 [Si(CH_3_)_2_], 18.66 [Si*C*(CH_3_)_3_], 26.0 [SiC(*C*H_3_)_3_], 35.22 (*C*H_2_CH_2_OH), 61.16 (CH_2_*C*H_2_OH), 108.38 (C-8), 112.53 (C-4a), 113.94 (C-3), 117.74 (C-6), 125.81 (C-5), 153.78 (C-8a), 155.62 (C-4), 159.66 (C-7), 161.86 (C-2). MS (EI, 70 eV): *m*/*z* (%) = 320 (M^+^, 45), 292 (20), 263 (100), 245 (70), 233 (35), 189 (35). Elemental anal. calcd. for C_17_H_24_O_4_Si: C, 63.72; H 7.55. Found: C, 63.60; H, 7.71.

**4-(2-Bromoethyl)-7-(*t*-butyldimethylsilyloxy)-2*H*-chromen-2-one (5):** A solution of **4** (320 mg, 1.00 mmol) and tetrabromomethane (730 mg, 2.20 mmol) in dry dichloromethane (10 mL) was stirred at 0 °C under a nitrogen atmosphere. A solution of triphenylphosphane (525 mg, 2.00 mmol) in dry dichloromethane (2 mL) was added slowly. After removing the ice bath, stirring was continued for 60 min. The mixture was concentrated under reduced pressure and the residue purified by column chromatography (dichloromethane/*n*-hexane, 8:1) to give 301 mg (79%) of solid product **5**; *R*_f_ 0.64; mp 88.5–89.4°C. IR (KBr): 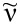 = 3428, 3065, 1590, 1438, 1184, 1120, 721, 696, 538 cm^−1^. ^1^H NMR (CDCl_3_, 500 MHz): δ = 0.25 [s, 6H, Si(CH_3_)_2_], 0.99 [s, 9H, C(CH_3_)_3_], 3.29 (t, *J* = 7.25 Hz, 2H, C*H*_2_CH_2_Br), 3.46 (t, *J* = 7.25 Hz, 2H, CH_2_C*H*_2_Br), 6.19 (s, 1H, 3-H), 6.80 (m, 2H, 6-H and 7-H), 7.44 (d, *J* = 9.14 Hz, 1H, 5-H). ^13^C NMR (CDCl_3_, 125 MHz): δ = −3.98 [Si(CH_3_)_2_], 13.68 [Si*C*(CH_3_)_3_], 25.97 [Si(*C*H_3_)_3_], 28.77 (*C*H_2_CH_2_Br), 35.21 (CH_2_*C*H_2_Br), 108.61 (C-8), 112.74 (C-4a), 113.13 (C-3), 117.83 (C-6, 125.15 (C-5), 152.48 (C-8a), 155.75 (C-4), 159.86 (C-7), 161.29 (C-2). MS (EI, 70 eV): *m*/*z* (%) = 384 (M^+^, 25), 327 (95), 302 (25), 245 (100), 217 (25), 189 (40), 115 (40). HRMS: calcd. for C_17_H_23_O_3_SiBr [M^+^+H+Na]: 405.0929; Found: 405.0847.

***t*-Butyl 4-{7-[(*t*-Butyldimethylsilyl)oxy]-2-oxo-2*H*-chromen-4-yl}-2-[diphenyl(methylidene)amino]butanoate (7):** A 25-mL two-necked flask equipped with a magnetic stirrer and a connection to a combined nitrogen/vacuum line was charged with the ester **6** (806 mg, 2.73 mmol) and closed with a septum. The air in the flask was replaced by nitrogen and dry THF (10 mL) was added. The mixture was cooled to −78 °C and a 1.6 M solution of *n*-BuLi in *n*-hexane (1.7 mL, 2.7 mmol) was injected by syringe. In a second flask, bromide **5** (615 mg, 1.61 mmol) dissolved in dry THF (2 mL) was added dropwise to the above-mentioned mixture with stirring at −78 °C. Stirring was continued at the same temperature for 2 h and then the mixture was allowed to warm up to r. t. over 1 h. A saturated aqueous solution of ammonium chloride (5 mL) was added. After three extractions with ethyl acetate (20 mL each) the combined organic layers were dried with sodium sulfate and concentrated under reduced pressure. The residue was purified by column chromatography (dichloromethane/ethyl acetate, 20:1) to give solid product **7** (645 mg, 67%); *R*_f_ 0.51; mp 191.8–192.4 °C. IR (KBr): 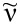 = 2929, 2856, 1714, 1616, 1393, 1286, 1245, 1154, 1088, 1000, 885, 701 cm^−1^. ^1^H NMR (CDCl_3_, 500 MHz): δ =0.25 [s, 6H, Si(CH_3_)_2_], 0.99 [s, 9H, SiC(CH_3_)_3_], 1.46 [s, 9H, OC(CH_3_)_3_], 2.23 (m, 2H, NCHC*H*_2_), 2.77 (m, 2H, NCHCH_2_C*H*_2_), 4.05 (m, 1H, NCH), 6.07 (s, 1H, 3-H), 6.75 (dd, *J* = 8.51 Hz, *J* = 2.21 Hz, 1H, 6-H), 6.78 (d, *J* = 2.52, 1H, 8-H), 7.56 (d, *J* = 8.5 Hz, 1H, 5-H), 7.14 (m, 2H), 7.35 (t, *J* = 7.57, 2H), 7.40 (m, 4H), and 7.69 (d, *J* = 7.57 Hz, 2H) (other aromatic H). ^13^C NMR (CDCl_3_, 125 MHz): δ = −3.98 [Si(CH_3_)_3_], 18.69 [Si*C*(CH_3_)_3_], 26.0 [SiC(*C*H_3_)_3_], 28.48 [OC(*C*H_3_)_3_], 32.81 (NCHCH_2_CH_2_), 65.31 (NCH), 81.93 [O*C*(CH_3_)_3_], 108.33 (C-8), 111.69 (C-4a), 113.77 (C-3), 125.96 (C-6), 128.53 (C-5), 139.67 (C-8a), 155.65 (C-4), 156.29 (C-7), 159.49 (C-2), 161.75 (C=N), 171.12 (C=O), 128.97, 129.19, 130.94, and 136.74 (other aromatic C). MS (EI, 70 eV): *m*/*z* (%) = 620 ([M+Na]^+^, 18), 598 ([M+H]^+^, 25), 542 (100), 208 (26). Elemental anal. calcd. for C_36_H_43_NO_5_Si: C, 72.33; H, 7.25; N, 2.34. Found: C, 72.18; H, 7.44; N, 2.17.

***rac*-(7-Hydroxycoumarin-4-yl)ethylglycine (1):** A solution of 7 (300 mg, 0.50 mmol) in 17% hydrochloric acid (10 mL) was stirred at r. t. for 24 h. The mixture was then repeatedly extracted with diethyl ether, until TLC of the extracts was found to be negative. The acid aqueous phase was lyophylized to give colorless, crystalline product 1 (79 mg, 60%) whose ^1^H NMR data were identical with those described in the literature [10]. ^13^C NMR (DMSO-*d*_6_, 125 MHz): δ = 28.95 (NCH*C*H_2_CH_2_), 31.07 (NCHCH_2_*C*H_2_), 51.88 (NCH), 102.86 (C-8), 109.91 (C-4a), 111.16 (C-3), 113.41 (C-6), 126.57 (C-5), 155.46 (C-8a), 160.67 (C-4), 161.72 (C-7), 169.27 (C-2), 170.93 (C=O). LC-MS: *m*/*z* (%) =264 ([M+H]^+^, 100).
